# Prevalence and risk factors of intestinal parasitic infections among preschool and school-aged children in Egypt: a systematic review and meta-analysis

**DOI:** 10.1186/s12889-025-23325-8

**Published:** 2025-06-10

**Authors:** Ahmed Azzam, Heba Khaled

**Affiliations:** 1 Department of Microbiology and Immunology, Faculty of Pharmacy, Helwan University, Cairo, Egypt; 2 Department of Biochemistry, Faculty of Pharmacy, Cairo University, Cairo, Egypt

**Keywords:** Intestinal parasite infections, IPIs, Preschool, School, Children, Egypt, Meta-analysis

## Abstract

**Introduction:**

Intestinal parasitic infections (IPIs) are a major public health concern, particularly among children in low- and middle-income countries, where limited resources and data hinder effective interventions. This meta-analysis consolidates current evidence on the prevalence of IPIs among preschool and school-aged children in Egypt, identifies key risk factors, and examines trends in prevalence over time.

**Methods:**

Six databases (African Journals Online, African Index Medicus, PubMed, Scopus, Google Scholar, and Web of Science) were systematically searched from January 1, 2010, to January 1, 2025. Studies conducted in Egypt on apparently healthy preschool or school-aged children were included if they reported IPIs prevalence or risk factors. A random-effects model was employed to estimate pooled prevalence or risk ratios. The meta-analysis was performed using the ‘meta’ package in R (version 4.4.1), with statistical significance set at *p* < 0.05.

**Results:**

This meta-analysis included 21 studies conducted between 2009 and 2021, involving 54,282 school and preschooler children from both Lower and Upper Egypt. The pooled prevalence of at least one IPI was 46.5% (95% CI: 40.5–52.5). Sensitivity analyses confirmed the robustness of the finding, with no evidence of publication bias. Meta-regression analysis revealed that the prevalence of at least one IPI remained consistent from 2009 to 2021. The most prevalent parasite was *Entamoeba* spp. (10.9%), followed by *Giardia duodenalis* (7.3%) and *Enterobius vermicularis* (4.9%). Less common parasites included *Schistosoma mansoni* (1.3%), *Ancylostoma duodenale* (1.0%), *Schistosoma haematobium* (0.6%), *Heterophyes heterophyes* (0.7%), *Trichuris trichiura* (0.5%), and *Fasciola* spp. (0.3%). Key risk factors included age 6–10 years (RR = 1.5), rural residence (RR = 1.4), low socioeconomic status (RR = 2.4), poor handwashing practices (RR = 2.1), consuming unwashed vegetables (RR = 1.5), and low maternal education (RR = 1.62).

**Conclusion:**

These findings highlight the substantial burden of IPIs among Egyptian preschool and school-aged children, with nearly half infected by at least one parasite. The consistently high prevalence from 2009 to 2021 underscores the urgent need to reevaluate current control measures and prioritize interventions targeting the high-risk groups identified in this study.

**Supplementary Information:**

The online version contains supplementary material available at 10.1186/s12889-025-23325-8.

## Introduction

Intestinal parasite infections (IPIs) represent a major public health concern, particularly in low- and middle-income countries (LMICs) [1]. School-aged children are especially vulnerable due to their underdeveloped immune systems and frequent exposure to contaminated soil and environments. Additionally, close contact in communal settings, such as schools, further increases the risk of transmission among this age group. IPIs in children can result in various adverse outcomes, including malnutrition, anemia, and impaired growth and development [1, 2]. Addressing these challenges requires targeted interventions, such as improving sanitation and implementing school-based deworming programs, to mitigate the far-reaching impacts of these infections.

The prevalence of IPIs shows significant global variation, with notable differences observed between countries. In Ethiopia, a meta-analysis involving 56,786 school and preschool children estimated an overall IPIs prevalence of 48% [3]. in Iran, a meta-analysis including 68,532 school and preschool children, reported a lower pooled prevalence of 38% [4]. In contrast, Nepal reported a significantly lower prevalence, with an overall rate of 20.4% observed between 2011 and 2015 [5]. Furthermore, a meta-analysis of 19 African countries reported a pooled prevalence of intestinal protozoan parasites among schoolchildren at 25.8%, with Northern Africa showing the highest prevalence (42.2%) and Southern Africa the lowest (18.6%) [6]. In contrast, a meta-analysis of 32 Asian countries found a lower pooled prevalence of intestinal protozoan parasites at 20.8% among schoolchildren [7]. However, some countries, such as Lebanon and Tajikistan, reported significantly higher rates at 85.1% and 83.6%, respectively [7]. These regional differences highlight the need for country-specific, targeted public health strategies tailored to the parasitology profile of each region.

In Egypt, parasitic infections remain a significant public health concern, particularly affecting vulnerable populations such as schoolchildren, where overcrowded conditions are prevalent. The country’s geographical location, climate, and socio-cultural practices create favorable conditions for the proliferation of various ecto- and endo-parasites. Egypt’s predominantly hot and arid climate provides optimal conditions for the life cycles of many parasites. The Nile River and its extensive irrigation canals serve as breeding grounds for waterborne parasites, offering habitats for intermediate hosts such as snails. Moreover, domestic animals and livestock often act as reservoirs for zoonotic parasites, further facilitating their transmission to humans.

In response, we conducted a systematic review and meta-analysis to evaluate the prevalence, examine trends over time, and identify risk factors associated with IPIs among preschool and school-aged children in Egypt. By synthesizing data from across the country, this study offers a comprehensive understanding of the parasitological landscape, highlights high-risk populations, and provides a foundation for evidence-based interventions aimed at improving public health outcomes.

## Methods

### Search strategy

A comprehensive literature search was conducted using six databases: African Journals Online, African Index Medicus, PubMed, Scopus, Google Scholar, and Web of Science. The search spanned from January 1, 2010, to January 1, 2025, ensuring the inclusion of up-to-date data and capturing current trends in IPIs among Egyptian children.

The search strategy, outlined in Table S1, was tailored to match the specific functionalities and criteria of each database. Detailed examples of the search strategies employed for Scopus and PubMed are provided in Table S2. This study adhered to the PRISMA (Preferred Reporting Items for Systematic Reviews and Meta-Analyses) guidelines [8], with the full PRISMA 27-item checklist presented in Table S3.

### Eligibility criteria

#### Inclusion criteria

Studies were included if they met the following criteria: (1) provided prevalence data on any IPI or associated risk factors; (2) were conducted in Egypt; (3) focused on apparently healthy preschool or school-aged children; and (4) were published between January 1, 2010, and January 1, 2025.

#### Exclusion criteria

Studies were excluded if they met any of the following criteria: (1) focused exclusively on specific subgroups, such as children with specific medical conditions or those in inpatient or outpatient settings; (2) were literature reviews, preprints, or conference abstracts.

### Study selection

A comprehensive set of references was assembled from the six databases, and duplicates were identified and removed using Zotero. H.K. conducted the initial screening of titles, followed by a more detailed screening of titles and abstracts. Subsequently, the full texts of potentially relevant articles were assessed for eligibility based on predefined criteria, and the reference lists of these eligible articles were also reviewed. Each step of the selection process was cross-verified by A.A., and any discrepancies were resolved through discussion and consensus between the two authors.

### Data extraction

Data extraction was performed by H.K. and subsequently cross-verified by A.A. For each included study, the following details were collected: last name of the first author, publication year, governorate, study period, total sample size, percentage of males, percentage of rural participants, diagnostic method, sampling method, age of the children, and data relevant for conducting a risk factors meta-analysis.

### Quality assessment

The quality of the included studies was assessed by H.K. using the Joanna Briggs Critical Appraisal Checklist for Prevalence Studies [9]. This assessment was later verified by A.A. The original checklist items are detailed in Table S4. The checklist comprehensively examined essential elements, such as the relevance of the sampling frame, the appropriateness of sampling techniques and sample size, the clarity of study setting descriptions, the thoroughness of data analysis, the application of validated methods for parasite identification, the consistency of parasite detection procedures, and the sufficiency of the response rate. A threshold score of 6 out of 9 was applied to categorize a study as being of fair quality.

### Data synthesis

A random-effects model utilizing the inverse variance weighting method was applied to calculate pooled prevalence rates or risk ratios, along with their corresponding 95% confidence intervals (CIs), based on the type of data analyzed. To enhance generalizability and reliability, only studies with at least three estimates were included in the meta-analysis. The random-effects model was chosen to account for heterogeneity among studies, which could arise from differences in populations, settings, and methodologies. Predefined subgroup analyses were conducted based on the type of intestinal parasite examined. Heterogeneity across studies was assessed using the I-squared (I^2^) statistic, with I^2^ values above 75% indicating substantial heterogeneity. To ensure the robustness of the results, sensitivity analyses were performed using a leave-one-out approach. Publication bias was evaluated using a funnel plot and Egger’s test, with a *p*-value greater than 0.05 suggesting no evidence of bias. Meta-regression was performed to analyze the temporal trends in the prevalence of IPI over time. All statistical analyses were conducted using R software (version 4.4.1), with a *p*-value below 0.05 considered statistically significant.

## Results

### Characteristics of the included studies

**Fig. 1:**
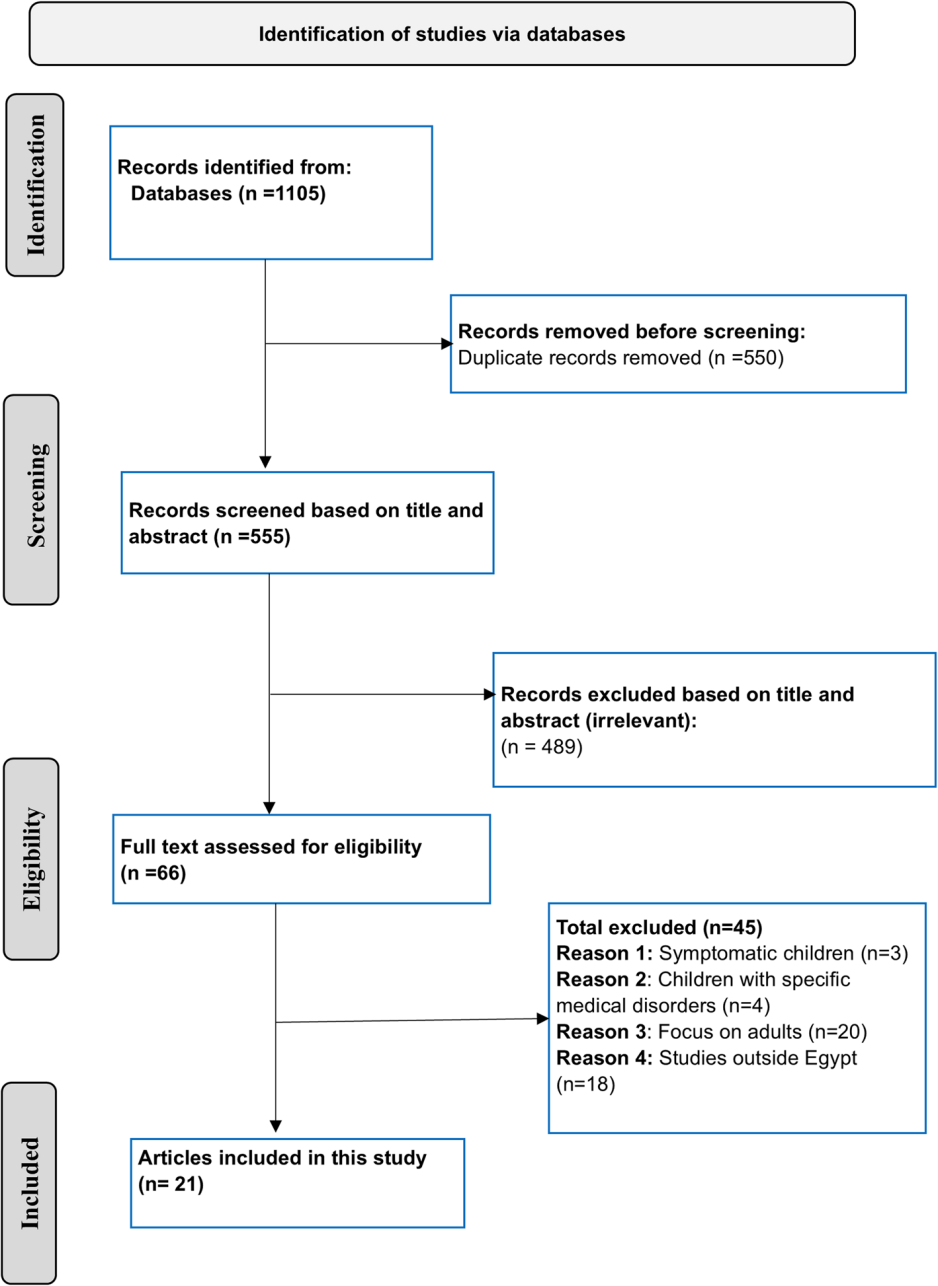
PRISMA flow diagram illustrating the selection process for the included articles

Out of the 1105 articles initially identified, 21 studies [10–30] met the inclusion criteria and were selected for analysis, as illustrated in Fig. 1. Collectively, these studies involved a total of 54,282 children. The research spanned a period of over a decade (2009–2021) and encompassed diverse geographic regions across both Lower and Upper Egypt. In Lower Egypt, the studied regions included Gharbia, El-Behera, Dakahlia, Damietta, Giza, Sharkia, Alexandria, Menoufia, Kafr El Sheikh, and Qalyubia. In Upper Egypt, the governorates analyzed were Sohag, Aswan, Minya, Assiut, Beni Suef, and Fayoum. Random sampling was the most commonly used approach, applied in 13 studies. Convenience sampling was utilized in 3 studies, while systematic random sampling and multistage sampling were each employed in 2 studies. Cluster sampling was used in 1 study. Regarding the quality of the studies, all included studies were of fair quality, scoring 6 or higher, as indicated in Table 1. Studies with lower scores typically had limitations such as a small sample size, unclear response rates, or an ambiguous study setting.

**Table 1:** Characteristics of the included studies

Last Name, Publication Year [Reference]	Governorate	Study period	Total sample size	Male %	Rural %	Diagnosis method	Sampling method	Children’s age (years)	Quality score (out of 9)
Elmonir, 2021 [10]	Gharbia	January–April 2018	996	48.4	52.8	Direct smear, FEC, Modified Kinyoun’s acid-fast staining	Random	1–15	8
Hegazy, 2014 [11]	El-Behera	NS	500	52.1	NS	Direct wet mount, FEC, Modified Ziehl–Neelsen staining	Random	2–6	7
Mohammad, 2012 [22]	Damietta	Oct 2011–Jan 2012	530	51.9	62.3	Direct wet mount, FEC, Cellophane tape smear	Random	4–18	9
Ahmed, 2022 [24]	Dakahlia	Oct 2020–Jan 2021	726	53	53.7	Direct wet mount, FEC, Kato-Katz, Cellophane tape	Cluster	6–18	9
Radwan, 2019 [25]	El-Behera	NS	810	71.4	NS	Direct smear, FEC	Random	6–17	8
Dyab, 2016 [26]	Aswan	Oct 2015–Mar 2016	300	56.7	66.7	Direct smear, FEC, Kato-Katz	Random	6–12	8
Yones, 2019 [27]	Assiut	2017–2018	630	47.3	100	Formalin-ethyl acetate, Zinc sulfate flotation, Modified Kinyoun’s staining	Random	6–17	9
Sherbini, 2013 [28]	Giza	2010–2013	650	64.6	100	Direct smear, FEC	Systematic random	5–15	9
Bayoumy (a), 2018 [29]	El-Behera	Oct 2016–Apr 2017	600	65.7	100	Direct smear, FEC, Flotation	Random	6–12	9
Bayoumy(b), 2018 [30]	Gharbia	Oct–Dec 2017	200	50	NS	Direct smear, Simple floatation, FEC	Random	6–12	8
Mahmoud, 2017 [12]	Dakahlia	Jan–Dec 2016	102	43.1	100	Direct wet mount, Formol-acetone concentration, Baermann, Modified acid-fast	Random	6–12	9
Nadi, 2017 [13]	Sohag	Jan–Dec 2015	200	51.5	50	Formalin-ethyl acetate, Modified Kinyoun’s staining	Random	6–12	9
Sakr, 2016 [14]	Damietta	Dec 2010–May 2011	200	53.5	NS	Direct smear, FEC	Systematic random	6–11	8
Ibrahium, 2011 [15]	Minia	Feb–May 2009	264	35.2	100	Direct wet mount, FEC	Random	6–12	7
Sharaf, 2021 [16]	Menoufia	February to May 2019	400	46.3	50	Direct smear, Kato-Katz, FECM, McMaster, Scotch adhesive tape	Multistage random	6–12	8
Farghly, 2014 [18]	Sharkyia	Jan–Dec 2013	859	NA	68	Direct smear, FES	Random	6–13	8
Abdel Fatah, 2012 [17]	Alexandria	Sep–Nov 2011	330	45.8	NS	Kato-Katz	Multistage random cluster	6–12	8
Haggag (a), 2018 [19]	Assiut, Bani Sweif, Fayoum, Menia, Sohag	Nov 2016–Mar 2017	30,083	72	NS	Urine filtration for *Schistosoma haematobium*	Convenience	6–16	8
Haggag (b), 2017 [20]	El-Behera, Dakahlia, Kafr El Sheikh, Qalyubia, Sharqia	Mar–Apr 2016	14,917	52	Mostly rural	Kato-Katz	Convenience	6–15	8
Allam, 2021 [21]	Alexandria	NS	400	54.5	100	Kato-Katz	Random	1–12	7
Naguib, 2018 [23]	El-Dakahlia, Gharbia, Damietta	Mar 2015–Apr 2016	585	50.6	56.8	PCR–RFLP, DNA sequencing for *Cryptosporidium* spp.	Convenience	≤ 8	8

*NS* Not Specified, *PCR–RFLP* Polymerase Chain Reaction—Restriction Fragment Length Polymorphism, *FEC* Formol-ether concentration

### Pooled prevalence of intestinal parasites among preschool and school-aged children in Egypt

**Fig. 2:**
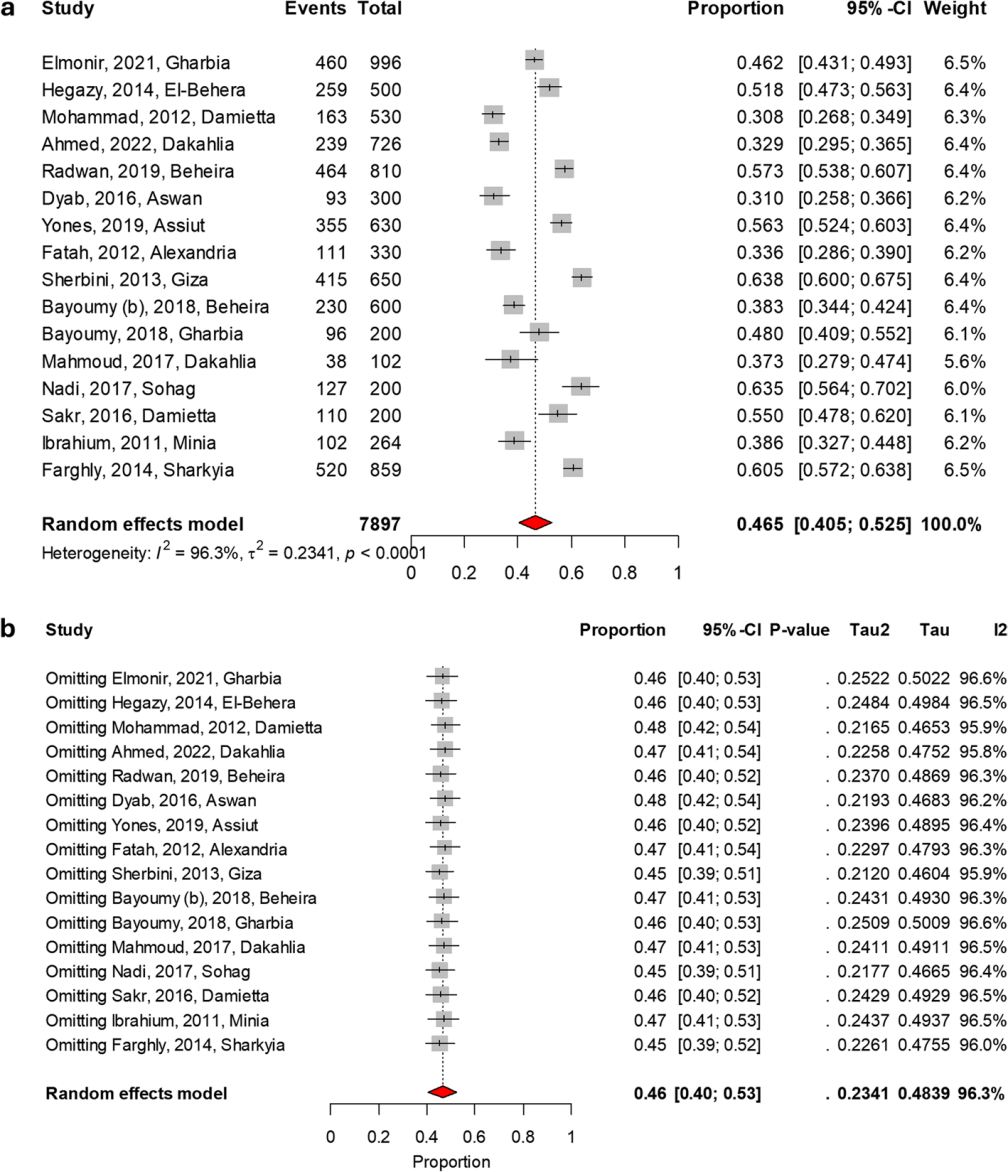
The prevalence of at least one parasitic infection among preschool- and school-aged children in Egypt. **a** Pooled prevalence under a random-effects model: 46.5% (95% CI 40.5–52.5). **b** Sensitivity meta-analysis demonstrating that the overall prevalence (46.5%) remained robust, with no more than a 2% variation upon removal of individual studies, indicating no major outliers

Table 2 presents the pooled prevalence along with the 95% confidence interval (CI), as well as the studies included in the analysis. The meta-analysis revealed that the pooled prevalence of children infected with at least one IPI in Egypt was 46.5% (95% CI 40.5–52.5, 16 studies), as shown in Fig. 2a. The sensitivity meta-analysis demonstrated that the overall prevalence (46.5%) remained robust, showing no more than a 2% variation when individual studies were removed, indicating no major outliers, as shown in Fig. 2b. Among the specific parasites identified, *Entamoeba* spp. had the highest prevalence at 10.9% (95% CI 7.7–15.2), followed by *Giardia duodenalis* with a prevalence of 7.3% (95% CI 4.7–10.9). *Enterobius vermicularis* showed a prevalence of 4.9% (95% CI 3.3–7.2), while *Ascaris lumbricoides* had a prevalence of 4.5% (95% CI 2.6–7.6). *Hymenolepis nana* exhibited a prevalence of 3.1% (95% CI 1.7–5.6), and *Cryptosporidium spp.* had a prevalence of 2.1% (95% CI 1.4–3.3). Additionally, *Schistosoma mansoni* had a prevalence of 1.3% (95% CI 0.7–2.4), and *Ancylostoma duodenale* was identified with a prevalence of 1.0% (95% CI 0.2–4.4). Less common parasites included *Schistosoma haematobium*, with a pooled prevalence of 0.6% (95% CI 0.2–1.9), *Heterophyes heterophyes* at 0.7% (95% CI 0.18–2.3), *Trichuris trichiura* at 0.5% (95% CI 0.1–2.3), and *Fasciola* spp. at 0.3% (95% CI 0.1–1.0). Overall, the heterogeneity (I^2^) was exceeding 75%.

**Table 2:** Pooled prevalence of intestinal parasites among school children in Egypt

Parasite	Prevalence (%)	95% Confidence Interval (CI)	Number of studies [References]
Overall Prevalence	46.5	40.5–52.5	16 [10–15, 17, 18, 22, 24–30]
*Entamoeba* spp.	10.9	7.7–15.2	15 [10–15, 18, 22, 24–30]
*Giardia duodenalis*	7.3	4.7–10.9	15 [10–15, 18, 22, 24–30]
*Enterobius vermicularis*	4.9	3.3–7.2	17 [10–15, 17, 18, 22, 24–31]
*Ascaris lumbricoides*	4.5	2.6–7.6	14 [10, 11, 13–15, 17, 18, 22, 24–29]
*Hymenolepis nana*	3.1	1.7–5.6	16 [10–16, 18, 22, 24–30]
*Cryptosporidium* spp.	2.1	1.4–3.3	5 [10, 12, 23, 26, 27]
*Schistosoma mansoni*	1.3	0.7–2.4	11 [12, 13, 16, 20–22, 24, 25, 28–30]
*Ancylostoma duodenale*	1.0	0.2–4.4	5 [10, 14, 25, 27, 28]
*Schistosoma haematobium*	0.6	0.2–1.9	3 [11, 19, 29]
*Heterophyes heterophyes*	0.7	0.18–2.3	3 [10, 25, 28]
*Trichuris trichiura*	0.5	0.1–2.3	3 [12, 18, 29]
*Fasciola* spp.	0.3	0.1–1.0	3 [10, 11, 25]

### Factors associated with intestinal parasites among preschool and school-aged children in Egypt

Table 3 presents the factors associated with intestinal parasites among preschool and school-aged children in Egypt, along with the I^2^ values. Younger age significantly increased susceptibility, with children aged 6–10 years showing an RR of 1.5 (95% CI: 1.2–1.7; *p* < 0.001) compared to those over 10 years of age. Living in rural areas was strongly correlated with a heightened risk of infection, with an RR of 1.4 (95% CI: 1.1–1.9; *p* < 0.001). Socioeconomic status emerged as a critical determinant, with children from low-income families facing a significantly greater risk compared to their high-income counterparts (RR = 2.4, 95% CI: 1.9–3.0; *p* < 0.001).

**Table 3:** Factors associated with intestinal parasite infections among preschool and school-aged children in Egypt

Factor	Included studies	Risk ratio	*P*-value	I^2^ value
(a) Significant Factors				
Younger age (6–10 years vs. > 10 years)	3 [24, 25, 27]	1.5 (95% CI: 1.2–1.7)	< 0.001	25.1
Living in rural areas vs. urban areas	3 [10, 24, 32]	1.4 (95% CI: 1.1–1.9)	< 0.001	83.0
Low socioeconomic status vs. high-income status	4 [10, 14, 24, 25]	2.4 (95% CI: 1.9–3.0)	< 0.001	0
Consumption of unwashed vegetables vs. washed vegetables	3 [10, 24, 28]	1.5 (95% CI: 1.2–2.0)	< 0.001	95.8
Poor handwashing practices vs. good practices	3 [10, 24, 28]	2.1 (95% CI: 1.1–4.0)	0.03	98.1
Low maternal education vs. high maternal education	3 [24, 25, 27]	1.62 (95% CI: 1.02–2.56)	0.04	90.1
(b) Insignificant Factors				
Low paternal education vs. high paternal education	3 [24, 25, 27]	1.2 (95% CI: 0.7–2.1)	0.5	93.1
Male gender vs. female gender	6 [10, 12, 14, 27, 28, 32]	1.2 (95% CI: 1.0–1.5)	0.054	85.8

Behavioral and dietary practices were additional contributing factors. The consumption of unwashed vegetables was associated with an increased risk of infection, reflected in an RR of 1.5 (95% CI: 1.2–2.0; *p* < 0.001). Similarly, poor handwashing practices significantly elevated the likelihood of infection, with an RR of 2.1 (95% CI: 1.1–4.0; *p* = 0.03). Furthermore, a low maternal education level was associated with an increased risk of IPIs in their children, with an RR of 1.62 (95% CI: 1.02–2.56; *p* = 0.04). In contrast, low paternal education was not significantly associated with an increased risk of IPIs (RR = 1.2, 95% CI: 0.7–2.1; *p* = 0.5). Male gender showed a marginal association with IPIs but did not reach statistical significance, with an RR of 1.2 (95% CI: 1.0–1.5; *p* = 0.054).

Additionally, zoonotic exposure was highlighted in two studies as a contributing factor [10, 32]. Poor fingernail hygiene was significantly associated with infection (*p* < 0.001) [24, 28].

### Meta-regression and publication bias testing

**Fig. 3:**
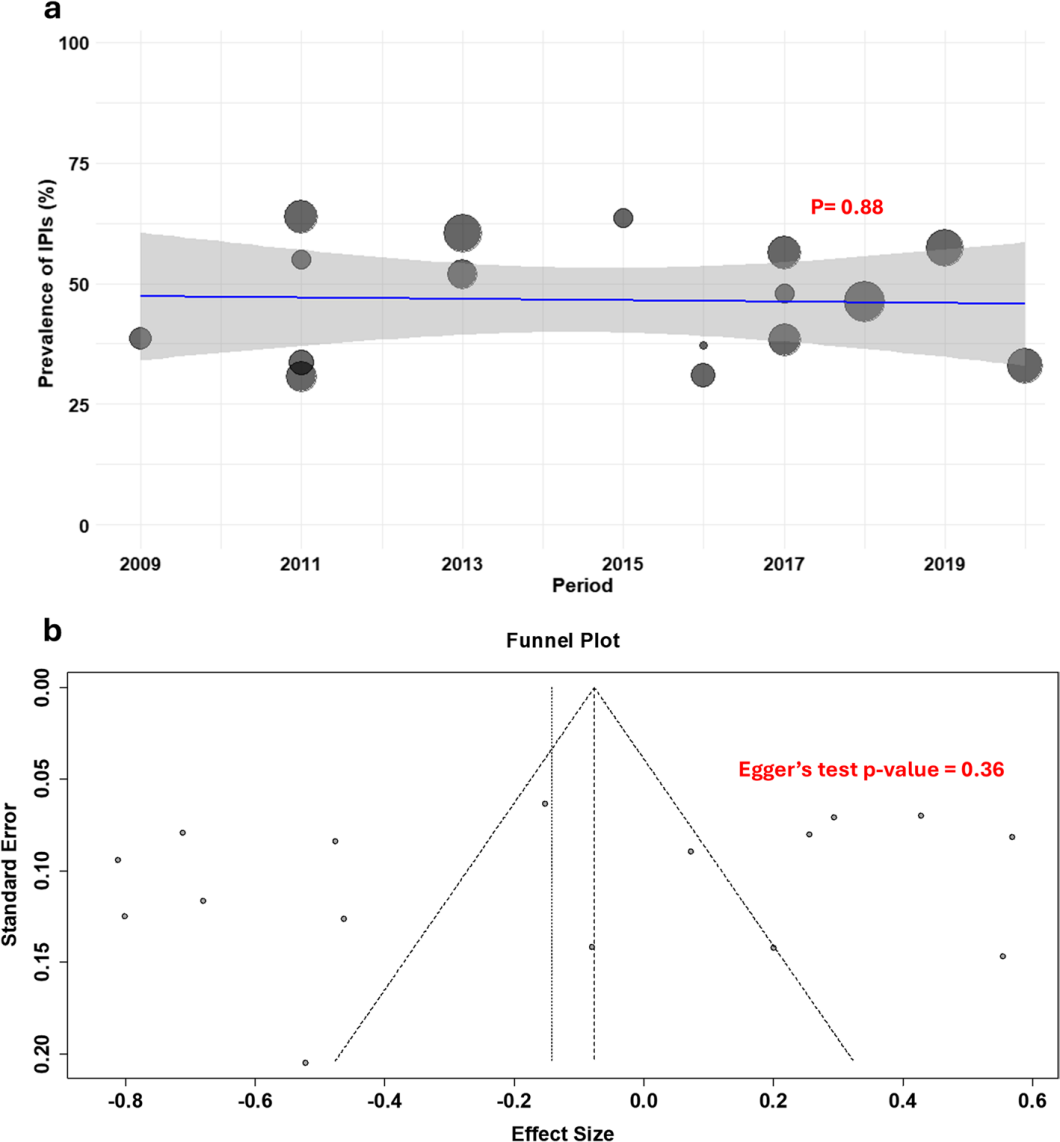
Meta-regression and publication bias testing analyses. **a** Meta-regression showing no significant association between study periods (2009–2021) and prevalence of at least one intestinal parasite infections (*p* = 0.817, *R*.^2^ = 0.4%), indicating stable prevalence over time. **b** Funnel plot showing no evidence of publication bias (Egger’s test, *p* = 0.36)

A linear regression model was applied to evaluate the relationship between the year (2009–2021) and the prevalence of at least one IPI. The analysis found no statistically significant association between year and prevalence (*p* = 0.88). The R^2^ value (0.4%) indicated that year explained less than 1% of the variation in prevalence, as shown in Fig. 3a. This suggests that the prevalence exhibited minimal variation and remained relatively consistent over that period. The publication bias assessment using a funnel plot indicated no evidence of publication bias, as supported by the Egger’s test with a *p*-value of 0.36, as shown in Fig. 3b.

## Discussion

The meta-analysis of 21 studies involving 54,282 preschool and school-aged children across Egypt revealed a high pooled prevalence of IPIs, with 46.5% (95% CI: 40.5–52.5) infected by at least one parasite. The prevalence remained stable from 2009 to 2021, as demonstrated by meta-regression analysis. *Entamoeba* spp. and *G. duodenalis* were the most prevalent parasites, at 10.9% and 7.3%, respectively. Less prevalent parasites included *S. mansoni* (1.3%), *A. duodenale* (1.0%), *S. haematobium* (0.6%), *H. heterophyes* (0.7%), *T. trichiura* (0.5%), and *Fasciola* spp. (0.3%). Key risk factors identified younger age (RR = 1.5), rural residence (RR = 1.4), low socioeconomic status (RR = 2.4), and poor hygiene practices, such as consuming unwashed vegetables (RR = 1.5) and inadequate handwashing (RR = 2.1). These findings underscore the significant burden of IPIs in Egyptian children and highlight the urgent need to reassess current control programs and implement targeted interventions that address socioeconomic disparities, promote hygiene education, and improve access to clean water, particularly in rural and underserved communities.

The pooled prevalence of IPIs in our study, covering the period from 2009 to 2021, was 46.5% (95% CI: 40.5–52.5%) among apparently healthy children. This high prevalence highlights the substantial proportion of asymptomatic carriers. These individuals may later progress to symptomatic stages and play a key role in sustaining transmission, particularly to vulnerable immunocompromised children who are at increased risk of developing more severe clinical outcomes. Furthermore, a recent systematic review indicates that IPIs in children under five years of age are strongly associated with adverse health outcomes, including stunting, wasting, underweight, undernutrition, and overall growth retardation [2]. Additionally, growing evidence suggests that IPIs, such as *G. duodenalis*, disrupt the gut microbiome which may also lead to post-infectious complications like IBS and chronic fatigue [33].

When compared to country-specific meta-analyses conducted among preschool and school-aged children during similar timeframes, this estimate is consistent with the prevalence reported in Ethiopia (2014 and beyond), which ranged from 42% (95% CI: 34%–49%) [3]. However, our findings indicate a higher prevalence than that documented among Iranian children (1996–2015), who exhibited a prevalence of 38% (95% CI: 33%–43%) [4], and Nepalese children, who demonstrated a markedly lower prevalence of 20.4% (95% CI: 15.04–26.25) during the period 2011–2015 [5]. Conversely, the prevalence is lower than that reported in Colombian children (2016–2020), which stood at 53% (95% CI: 37%–69%) [34]. Notably, all these meta-analyses demonstrated a downward trend in the prevalence of IPIs, as evidenced by subgroup analyses [3–5] or joinpoint regression analysis [34]. In contrast, our study revealed that the prevalence of IPIs remained relatively stable over 2009 to 2021 (*p* = 0.817). This indicates that existing control measures may not have been sufficient to reduce the burden over time. This calls for a reevaluation of current strategies and the implementation of more robust, evidence-based interventions.

In this study, *Entamoeba* spp. and *G. duodenalis* were identified as the most prevalent parasites, with pooled prevalence rates of 10.9% and 7.3%, respectively. These findings align with a meta-analysis on the prevalence of intestinal protozoan parasites among schoolchildren in Africa, which reported *Entamoeba* histolytica/dispar at 13.3% and Giardia spp. at 12% as the most common pathogenic parasites [6]. In contrast, a meta-analysis focusing on intestinal protozoan parasites among schoolchildren in Asia found that *G. duodenalis* was the most prevalent species, with a prevalence rate of 8.2% [7]. Additionally, our findings revealed that the pooled prevalence of *S. mansoni* and *S. haematobium* was relatively low, at 1.3% and 0.6%, respectively. Egypt has historically borne one of the highest global burdens of schistosomiasis, with *S. mansoni* and *S. haematobium* widely prevalent in different regions. To address this ongoing public health issue, Egypt’s Ministry of Health and Population launched the National Schistosomiasis Control Program in 1977 [35]. As a result of the implementation of mass treatment with praziquantel, combined with snail control and educational campaigns, the disease burden significantly decreased from over 30% in 1983 to less than 1% in 2010 [35]. These findings underscore the effectiveness of sustained governmental efforts and intervention programs in combating schistosomiasis, highlighting the success of long-term public health initiatives. Regarding the pooled prevalence of *Fasciola* spp., it was relatively low at 0.3%, in contrast to earlier studies conducted in Egypt during the 1990 s, which reported a prevalence of 3.0% to 5.0% among school-aged children using the Kato-Katz thick-smear technique [36, 37]. This difference reflects a continuous decline in the prevalence of *Fasciola* infections over time, likely due to improved control measures, public health interventions, and increased awareness in endemic regions.

Few meta-analyses have reported pooled RR for IPIs among school-aged children, all of which originate from studies conducted in Ethiopia [38–40]. These studies emphasize rural residence, lack of formal maternal education, irregular handwashing practices, failure to wash fruits and vegetables, and other poor hygiene practices as significant contributors to IPIs [38–40]. In this study, key risk factors for IPIs among preschool and school-aged children in Egypt include younger age, rural residence, low socioeconomic status, low maternal education, and poor hygiene practices. Younger children aged 6–10 years (RR = 1.5) are particularly vulnerable due to their immature immune systems and behaviors such as playing outdoors and frequent hand-to-mouth contact, which increase their exposure to contaminated environments. Rural residence (RR = 1.4) further elevates the risk, as these areas often lack access to clean water, proper sanitation, and healthcare services. Additionally, exposure to untreated water and agricultural waste in rural settings increases the likelihood of parasite transmission. Low socioeconomic status (RR = 2.40) exacerbates these risks, as families in such conditions often face overcrowded living environments, inadequate resources for proper hygiene, and limited access to healthcare. Poor hygiene practices, such as consuming unwashed vegetables (RR = 1.5) and insufficient handwashing (RR = 2.1), also contribute significantly to the spread of parasites. Unwashed vegetables, particularly those irrigated with contaminated water, can carry parasitic eggs or cysts, while inadequate handwashing after using the toilet or before handling food facilitates transmission. Together, these factors create a high-risk environment for IPIs in this population.

Additionally, Low maternal education level was significantly associated with an increased risk of IPIs in children (RR = 1.62). This finding highlights the critical role of maternal knowledge in childcare and hygiene practices. In contrast, low paternal education did not show a significant association with IPIs (RR = 1.2, 95% CI: 0.7–2.1; *p* = 0.5), possibly reflecting traditional roles in many societies where fathers are less directly involved in daily caregiving and hygiene practices. In light of these findings, public health interventions should prioritize improving access to clean water and sanitation in rural areas, implementing hygiene education programs with a focus on enhancing maternal knowledge, and promoting proper handwashing practices. Furthermore, addressing socioeconomic disparities are essential measures to effectively mitigate risk factors associated with intestinal parasite infections.

### Strengths and limitations

This analysis has several notable strengths. It provides a comprehensive overview of the parasitology profile among children in Egypt, offering valuable insights into the prevalence, trends, and associated risk factors for intestinal parasitic infections. The absence of publication bias, the robustness of pooled estimates confirmed through sensitivity analyses, and the fair quality of the included studies collectively strengthen the consistency and reliability of the findings. Nonetheless, several limitations must be acknowledged. First, the lack of prevalence data from all regions in Egypt restricts the generalizability of the findings to the entire country. Second, the lack of adequate prevalence data for certain parasites reduces the reliability of the pooled estimates and limits the ability to accurately assess temporal trends. These limitations highlight the urgent need for further research, particularly in regions with insufficient or no data. Addressing these gaps will be essential to enhancing our understanding of IPIs and informing targeted public health interventions.

## Conclusion

This study reveals a significant prevalence of IPIs among preschool and school-aged children in Egypt, with nearly half affected by at least one parasite. The consistently high rates observed between 2009 and 2021 emphasize the critical need to reassess existing control strategies. Develop targeted interventions for the most vulnerable groups identified in the analysis, including boys aged 6–10 living in rural areas. Additional focus should be placed on families with low socioeconomic status, limited maternal education, and poor hygiene practices.

## Supplementary Information

Supplementary Material 1.

## Data Availability

All data generated and analyzed throughout this study were included either in this article or its supplementary information file.

## Abbreviations

IPIs: Intestinal Parasite Infections
LMICs: Low- and Middle-Income Countries
PRISMA: Preferred Reporting Items for Systematic Reviews and Meta-Analyses
CIs: Confidence Intervals
PCR–RFLP: Polymerase Chain Reaction - Restriction Fragment Length Polymorphism
FEC: Formol-Ether Concentration
